# Extreme Ultraviolet Fractional Orbital Angular Momentum Beams from High Harmonic Generation

**DOI:** 10.1038/srep43888

**Published:** 2017-03-10

**Authors:** Alex Turpin, Laura Rego, Antonio Picón, Julio San Román, Carlos Hernández-García

**Affiliations:** 1 Departament de Física, Universitat Autònoma de Barcelona, E-08193 Bellaterra, Spain; 2 Center of Advanced European Studies and Research, 53175 Bonn, Germany; 3 Grupo de Investigación en Aplicaciones del Láser y Fotónica, Departamento de Física Aplicada, University of Salamanca, E-37008, Salamanca, Spain

## Abstract

We investigate theoretically the generation of extreme-ultraviolet (EUV) beams carrying fractional orbital angular momentum. To this end, we drive high-order harmonic generation with infrared conical refraction (CR) beams. We show that the high-order harmonic beams emitted in the EUV/soft x-ray regime preserve the characteristic signatures of the driving beam, namely ringlike transverse intensity profile and CR-like polarization distribution. As a result, through orbital and spin angular momentum conservation, harmonic beams are emitted with fractional orbital angular momentum, and they can be synthesized into structured attosecond helical beams –or “structured attosecond light springs”– with rotating linear polarization along the azimuth. Our proposal overcomes the state of the art limitations for the generation of light beams far from the visible domain carrying non-integer orbital angular momentum and could be applied in fields such as diffraction imaging, EUV lithography, particle trapping, and super-resolution imaging.

Light beams exhibit two intrinsic degrees of freedom associated to angular momentum: spin angular momentum (SAM) or polarization, indicating the direction in which the field oscillates; and orbital angular momentum (OAM) related to the spatial profile of the phase of the electric wave. Helical phased beams, also called optical vortices, exhibit a transversal spiral-phase structure or twist around the beam axis, thus having a well-defined OAM, which is characterized by the topological charge, 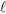, i.e. the number of 2*π*-phase shifts along the azimuth of the light beam. OAM beams, typically produced in the visible (VIS) and near-infrared (NIR) domains, are of special interest due to their additional degree of freedom over the control of light beams, making them suitable in different fields such as optical communication, micromanipulation, phase-contrast microscopy, or quantum optics^1^,^2^.

Interestingly, it has been recently shown that light beams can carry fractional or non-integer OAM, i.e., photons can exhibit a half-integer angular momentum^3^,^4^,^5^,^6^,^7^,^8^,^9^,^10^. In this sense, conical refraction (CR) produced with optically biaxial crystals has been demonstrated to be an efficient method to generate half-integer OAM beams^11^,^12^,^13^,^14^,^15^. CR beams exhibit a rotating linear polarization along the azimuth as a consequence of their fractional-OAM^11^. Applications of CR beams exhibiting fractional-OAM have been reported in areas such as optical trapping, free-space optical communications, material processing, and super-resolution imaging, see ref. 16 and references therein. These applications are limited to the VIS-NIR domain due to the spectral bandwidth at which biaxial crystals are transparent (typically 400 nm–3000 nm). Therefore, new generation mechanisms of fractional-OAM beams at shorter wavelengths –such as the EUV and soft x-rays– are needed to extend the applicability of these techniques to the nanometer scale.

In principle, non-linear optical processes could be used to up-convert fractional-OAM beams into shorter wavelength regimes. For the case of CR, perturbative second harmonic generation (SHG) has been exclusively studied so far. However, since in SHG only *oo* → *e* and *oe/eo* → *e* processes are allowed –where *o* and *e* stand for the ordinary and extraordinary polarization, respectively– the output SHG beam necessarily looses the non-homogeneous polarization distribution carried by the CR beam at the fundamental frequency^17^. As a consequence, perturbative harmonic generation does not up-convert the CR beam structure into higher frequencies, preventing the extension of half-integer OAM beams into shorter wavelength regimes^18^.

High-order harmonic generation (HHG) is known as a unique non-perturbative frequency up-conversion process for the generation of coherent EUV and soft x-ray radiation, emitted in the form of attosecond bursts^19^,^20^. The underlying physics at the microscopic level can be simply understood by the so-called *three-step model*^21^,^22^: an electron is tunnel ionized from an atom or molecule by an intense linearly polarized laser field, then accelerated, and finally driven back to its parent ion, releasing all the energy acquired during the process in the form of high-order harmonics upon recombination, extending from the EUV to the soft x-ray regimes^23^. From the macroscopic point of view of HHG, an infrared laser beam is focused into a gas target, and, if efficient phase-matching conditions are met^24^, an EUV/x-ray beam is efficiently emitted.

A remarkable aspect of HHG is its fully coherent nature, mapping the characteristics of the driving field to the high-frequency spectral region. This property allows to harness the angular momentum (OAM and SAM) characteristics of the harmonic radiation through modifications of the driving field. Recently, after a first experiment of OAM-HHG^25^, it has been shown that OAM conservation in HHG leads to the generation of several highly charged –single OAM– EUV vortex beams from a NIR vortex beam^26^,^27^,^28^,^29^. Noticeably, the OAM content of each EUV vortex beam is not limited to a single value, and it can be increased thanks to the non-perturbative behavior of HHG^30^. On the other hand, although the efficiency of the HHG process is very sensitive to the ellipticity of the driving field^31^, different schemes have recently succeeded in generating elliptically and circularly polarized harmonics^32^,^33^,^34^,^35^ and attosecond pulses^36^,^37^,^38^, thus controlling the SAM of EUV/soft x-ray harmonics. As a consequence, HHG stands as a good candidate to translate the fractional-OAM and polarization properties of CR beams to the EUV and soft x-ray domains. However, althoguh OAM^26^,^30^ and SAM^32^,^39^ haven been recently proven to be conserved separately in HHG, CR-driven HHG opens a new scenario where both SAM and OAM have to be taken into account. Whether spin-orbit interactions^40^ may modify the conservation rules in HHG remains unobserved.

In this work, we perform a theoretical analysis of the generation of EUV fractional-OAM beams through high-order harmonic generation driven by conical refraction beams. In contrast to the perturbative SHG process, HHG retains the ring-like transverse intensity profile and polarization distribution of CR beams, thus enabling the generation of fractional-OAM beams in the EUV/soft x-ray regimes. We characterize the spatio-temporal structure of the EUV harmonics. We demonstrate that OAM and SAM conservation rules restrict the emission of *q*-th order harmonic to highly charged fractional-OAM, 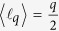. The similar divergence of the emitted harmonics allows for the synthesization of structured helical attosecond beams –or “structured attosecond light springs”– that preserve the polarization structure and transverse intensity pattern of the fundamental beam.

## Physical Scenario: high-order harmonic generation driven by conical refraction beams

**Figure 1:**
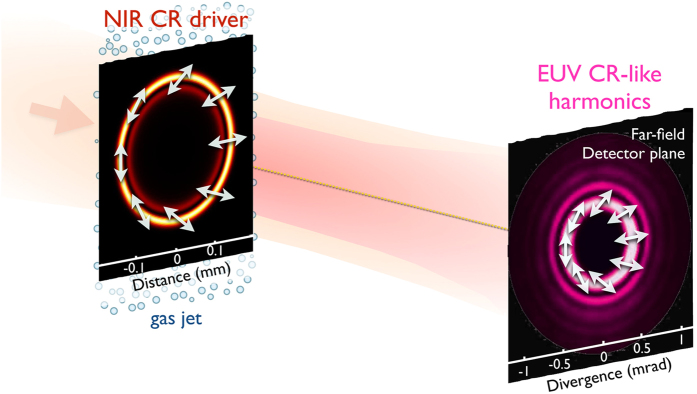
Scheme to generate EUV high-order harmonics from conical refraction (CR) beams: an intense NIR CR beam generated with a biaxial crystal is focused onto a gas jet. The left inset shows the NIR CR beam intensity distribution at the focal plane. The linear polarization distribution (depicted with white arrows) rotates along the azimuth. High-order harmonics are generated in each atom of the gas jet, and then propagated towards a far-field detector. The right inset shows the far-field HHG intensity and polarization distributions, which retain the CR-like beam structure.

The physical scenario to produce fractional-OAM EUV beams is sketched in Fig. 1. A NIR CR beam, characterized by a double ring transverse intensity profile and rotating linear polarization along the azimuth (left inset), is focused into a gas jet. High-order harmonics are generated upon interaction in the gas, and propagated to a far-field plane where they are detected (right inset).

Conical refraction occurs when a light beam propagates through a biaxial crystal parallel to one of the two optic axes^16^,^41^,^42^. A focused Gaussian input beam under conditions of CR is transformed into a pair of concentric bright rings split by a dark ring at the otherwise expected focal plane^42^,^43^,^44^. The state of polarization of the rings is linear at every point with the azimuth rotating continuously along them, preserving orthogonal polarizations between diametrically opposite points on the rings, as shown by double arrows in the left hand side of Fig. 1. This polarization distribution only depends on the orientation of the plane of the crystal optic axes^45^. A more detailed analysis of the CR beam (see Methods) shows that it is composed of two components: the *B*_0_ beam, with null OAM (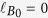), and the *B*_1_ beam, with one unit of OAM [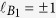, the ± sign depending on whether the input beam to the crystal is right (+) or left (−) handed circularly polarized]. Both components are orthogonally circularly polarized, so their photons exhibit a definite SAM of 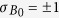 and 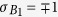, respectively. As a result, the fundamental beam has an equal number of photons from the *B*_0_ and *B*_1_ beams, thus exhibiting linear polarization, 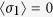, and fractional-OAM, 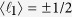. In the results presented below, we restrict to the case of right handed circularly polarized input beams. With respect to the dimensions of the light rings, common biaxial crystals allow for feasible radii in the range of 16–500 μm (see Methods). Note that the generated CR rings can be further (de)magnified with the use of additional imaging lenses after the biaxial crystal to diffracted-limited CR rings down to *R*_0_ = 5 μm (*R*_0_ representing the radius of the dark ring of the CR beam). It is worth mentioning that the position of the biaxial crystal is independent for the generation of the CR rings, as long as it is placed before the focal plane of the input beam. This is useful in situations where extreme high powers are needed, such as in the case of HHG, in order to keep intensity conditions below the damage threshold of the crystal (of 3 GW/cm^2^ in KGd(WO_4_)_2_^45^). We note that the current crystal technology provides crystals with 10 mm × 10 mm input faces, which would allow using fundamental IR beams with a beam waist radius of *w*_0_ ≈ 5 mm (previous to focusing), allowing a maximum input power of about 2.35 GW. Note that in the simulations presented here the laser power is of about 1 GW, well below the damage threshold of the crystal. Therefore, our method can be feasibly experimentally implemented with the current technology. On the other hand, the spectral bandwidth in the visible domain tolerated by the crystal is of Δ*λ* = 100 nm due to dispersion^46^, which causes a dependence between the direction of the optic axis and the wavelength of the input beam. This restricts the use of short pulses down to Fourier limited ≈8.8 fs at full-width-half-maximum (FWHM) for a central wavelength *λ*_0_ = 800 nm. Note however that the allowed pulse duration could be shortened by using a pair of compensation prisms, which extends the spectral bandwidth to 250 nm^47^, allowing the use of single-cycle pulses (2.7 fs) at 800 nm.

In order to study HHG driven by CR beams, we perform numerical simulations including propagation through the electromagnetic field propagator^48^ (see Methods). The NIR driving beam is modeled as a CR beam with a waist radius of *w*_0_ = 15 *μ*m and *R*_0_ = 10*w*_0_. In the left inset of Fig. 1 we present the transverse intensity profile of the input beam at the focal plane (*z* = 0). The driving pulse is considered to have 7.7 fs FWHM in duration, *λ*_0_ = 800 nm as central wavelength, and a maximum peak intensity at focus of 1.4 × 10^14^ W/cm^2^. Note that the pulse duration is within the limit of that tolerated by the crystal bandwidth. Longer pulses –which were not implemented due to high computational time– could be used without any substantial modification of the physics presented here. High-order harmonics are generated in a 200 *μ*m-thick argon gas jet, and propagated towards a far-field detector. The thickness of the gas jet has been chosen to ensure that the CR double ring structure is maintained during the whole light-matter interaction region, (see Methods).

## Results and Discussion

### High-order harmonic beams exhibiting fractional orbital angular momentum

**Figure 2:**
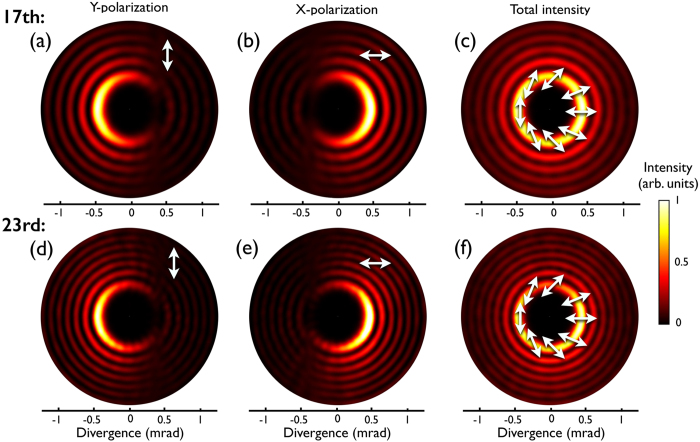
Angular intensity plots showing the spatial far-field profile of the 17^th^ harmonic (47.1 nm, 26.4 eV, first row) and the 23^rd^ harmonic (34.8 nm, 35.6 eV, second row). First and second column show, respectively, the projection over the vertical (*y*) and horizontal (*x*) polarization directions. Third column shows the sum of both components, where the linear polarization distribution is depicted with white arrows.

In Fig. 2 we present the simulated angular intensity profiles of the 17^th^ harmonic (47.1 nm, 26.4 eV, first row) and the 23^rd^ harmonic (34.8 nm, 35.6 eV, second row), projected into the y-polarization (a,d), x-polarization (b,e), and the sum of both components (c,f). As indicated in Fig. 2(c,f) both harmonics are linearly polarized in a direction that rotates along the azimuth as that of the fundamental CR beam. Regarding the intensity profile, it is clear that the on-axis nodal point of the fundamental beam is preserved in the far-field emission of harmonics. We note that only the outer ring of the fundamental CR beam is intense enough to drive HHG. Thus, upon far-field propagation, the harmonics present a slightly different intensity profile from the fundamental, with an intense inner ring and weaker outer rings. Regarding the far-field EUV yield of the CR-like harmonic beams, our simulation results indicate that the efficiency is similar to that presented in HHG driven by Gaussian beams, where up-conversion efficiencies up to 10^−5^ can be achieved if phase-matching conditions are met^23^,^49^,^50^,^51^.

**Figure 3:**
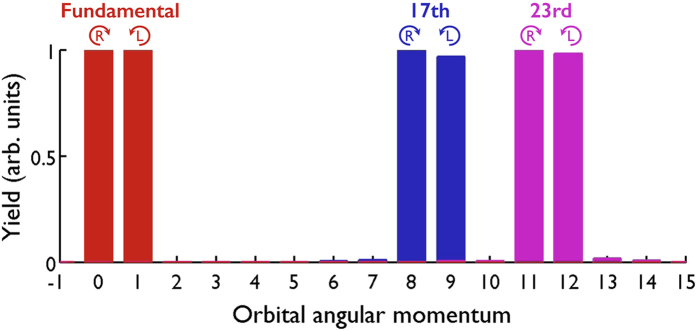
OAM spectrum of the fundamental beam (red bars), the 17^th^ harmonic (blue bars) and the 23^rd^ harmonic (pink bars). The two components of the fundamental CR beam are clearly distinguished: the *B*_0_ beam (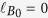, 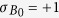), and the *B*_1_ beam (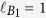, 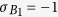). Thus, the fundamental beam exhibits fractional-OAM 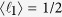. The high-order harmonics present analogously two OAM contributions with counter-rotating polarization, corresponding to the R and L absorption channels. Angular momentum conservation conveys the generation of fractional-OAM harmonics: 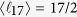, and 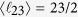. Note that the OAM spectra are performed over the projections over the *y* polarization (those over the *x* polarization give similar results). The OAM spectrum of each beam results from the integration along the divergence angle, and each one is normalized independently.

In order to study the OAM content of the harmonic beams, in Fig. 3 we plot the OAM spectrum of the fundamental CR beam (red bars), the 17^th^ harmonic (blue bars) and the 23^rd^ harmonic (pink bars). The OAM spectrum is obtained by performing the Fourier Transform of the field along the azimuth coordinate, thus, giving a clearer sense of the OAM content rather than looking directly into the spatial phase distribution. We can observe that the fundamental CR beam is composed of two components, the *B*_0_ beam (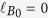, 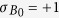), and the *B*_1_ beam (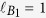, 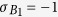) with equal yield, thus exhibiting linear polarization, 〈*σ*_1_〉 = 0, and fractional-OAM, 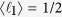.

During the HHG process, OAM^26^,^30^ and SAM^32^,^39^ have been shown to be conserved separately. However, HHG driven by CR beams opens a new scenario where conservation of both OAM and SAM have to be taken into account together. From the photon picture one can consider the generation of the *q*-th order harmonic from the absorption of *n*_0_ and *n*_1_ photons from the *B*_0_ and *B*_1_ beams respectively, conveying the absorption channel (*n*_0_, *n*_1_). Let us analyze separately the conservation rules that apply in our scheme of CR-driven HHG. Energy conservation implies *q* = *n*_0_ + *n*_1_. Moreover, we note that parity conservation forces *n*_0_ + *n*_1_ to be an odd integer while the restriction over the photon spin (|σ| = 1) implies that *n*_1_ = *n*_0_ ± 1, i.e., the number of photons absorbed from the *B*_0_ and *B*_1_ beams only differs by one. As a consequence, there are only two possible channels to generate the *q*-th order harmonic: the R channel (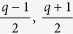), and the L channel (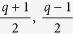). SAM conservation of each channel leads to


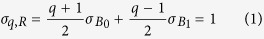



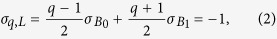


where one can identify the notation of each channel with its resulting polarization, right circular (R) or left circular (L). On the other hand, OAM conservation of each separate channel implies the following build-up rules


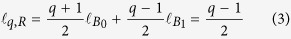



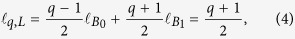


i.e., the OAM content of the *q*-th order harmonic is limited to two contributions, 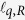 and 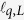. Finally, note that the efficiency of the HHG process in a single-color scheme –as that presented in this work– decreases dramatically with the ellipticity of the driving beam^31^, thus restricting HHG to linearly polarized drivers. Therefore, both channels R and L are forced to be present with similar efficiency, yielding to a net absorption of linearly polarized photons. As a consequence, the resulting *q*-th order harmonic exhibits linear polarization and fractional-OAM,


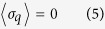



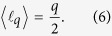


In Fig. 3 we observe that the 17^th^ (blue) and 23^rd^ (pink) harmonics present two OAM contributions with counter-rotating polarization, corresponding to the R and L channels described previously. As a consequence, angular momentum conservation implies that the 17^th^ harmonic exhibits fractional-OAM of 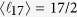, and the 23^rd^ 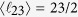, as predicted above. This result shows that OAM and SAM are not exchanged in CR-driven HHG, but conservation of both quantities separately leads to the generation of fractional-OAM harmonics.

### Structured attosecond helical beams

HHG offers the exciting perspective of synthesizing EUV/soft x-ray pulses of attosecond duration. An attosecond pulse train is obtained by the selection of the higher frequency part of the HHG spectrum. This synthesis is possible because the high-order harmonics exhibit similar intensity (like a frequency comb), phase, and divergence. Recent results showed that EUV harmonic vortices with single-OAM exhibit similar divergence due to OAM conservation^26^,^28^,^29^, thus allowing the synthesization of harmonic vortices into attosecond helical beams, i.e., attosecond pulse trains delayed along the azimuthal coordinate. This particular spatio-temporal light structure, also named “light spring”, results from the addition of harmonic vortices with different topological charges^52^.

**Figure 4:**
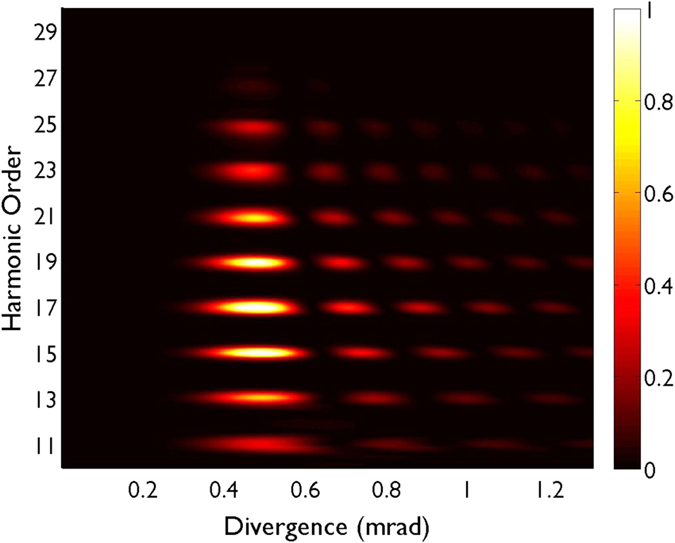
Angular intensity profile of the high-order harmonics driven by CR beams (linear scale, arb. units). All the high-order harmonics (from the 11-th to the 25-th) present a prominent ring profile with divergence around 0.5 mrad.

In Fig. 4 we present the spatial intensity divergence (linear scale) of the high-order harmonics produced in our HHG scheme driven by CR beams. All the high-order harmonics presented (from the 11-th to the 25-th) present similar intensity and divergence, exhibiting a ring profile of about 0.5 mrad. Thus, fractional-OAM harmonics preserve the similar divergence also shown by integer-OAM harmonics.

**Figure 5:**
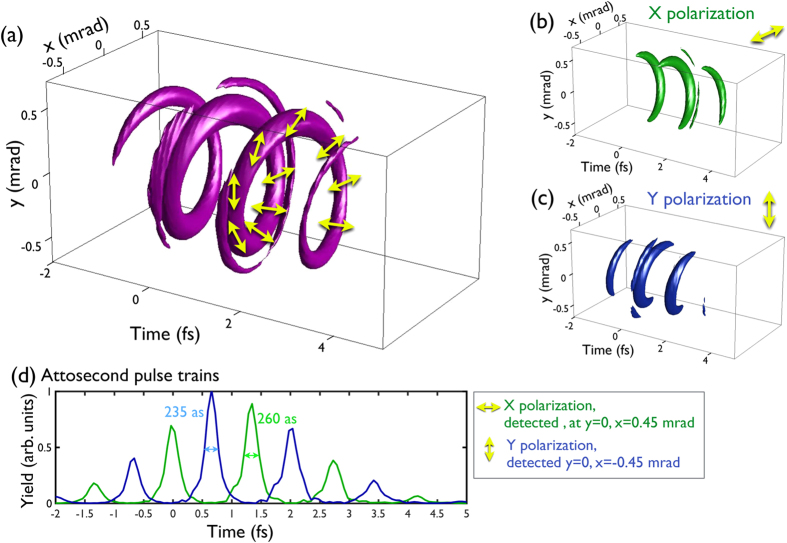
Structured helical attosecond beam obtained after Fourier transforming the 3D fractional OAM HHG spectrum and filtering the low-order harmonics (below the 11-th). Panel (a) plots an isosurface of the total intensity (where the polarization is depicted with yellow arrows) and panels (b,c) show the projections over the horizontal (*x*) and vertical (*y*) polarization directions respectively. A single attosecond helix with rotating polarization along the azimuth is obtained, representing one of the most complex field structures with unique spatio-temporal resolution. Panel (d) shows the attosecond pulse trains detected at the far-field spatial positions with pure horizontal (green, *y* = 0, *x* = 0.45 mrad) and vertical (blue, *y* = 0, *x* = −0.45 mrad) polarization.

In Fig. 5 we present the helical attosecond beam obtained after Fourier transforming the 3D fractional OAM harmonic spectrum, where we have filtered out the low-order harmonics (below the 11-th), simulating the transmission of the harmonic radiation through an aluminum filter. Panel (a) plots an isosurface of the total intensity, where the polarization is depicted with yellow arrows, and panels (b,c) show the projections over the horizontal (*x*) and vertical (*y*) polarization directions respectively. Panel (d) shows the attosecond pulse trains detected at the far-field spatial positions with pure horizontal (green) and vertical (blue) polarization. We observe that the attosecond beam is composed of a pulse train –with pulses of ~250 attoseconds FWHM– delayed along the azimuth. As a result, an structured attosecond helical beam –or “structured attosecond light spring”– is obtained, where the linear polarization rotates along the azimuth coordinate. Note that the number of interwinded helices that conform the attosecond helical beam is given by 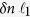, i.e., the order difference between successive harmonics in the spectrum (*δn*) times the topological charge of the driving beam (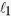)^52^. While in integer-OAM HHG two interwinded helices were obtained^26^,^28^,^29^, when using fractional-OAM beams a single helix is produced (note that 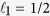 and *δn* = 2, as only odd-order harmonics are produced).

We expect that macroscopic phase matching conditions –such as the relative position between the focus and the gas jet, the gas jet density and geometry, etc.– may offer the possibility to control the spatial and temporal properties of the structured attosecond helical beams in a similar way as it was recently predicted for non-structured helical beams^28^. These “structured attosecond light springs” represent one of the most complex field structures presented to date, and exhibit unique properties for the inspection of spatio-temporal dynamics at the nanometer and attosecond scales in polarization dependent systems, such as molecules.

## Conclusions

In conclusion, we have theoretically demonstrated the generation of EUV beams carrying fractional OAM through HHG. To do so, we have considered a fs CR beam in the NIR domain focused into an argon gas target at standard experimental conditions. HHG and harmonic phase-matching are demonstrated to preserve the CR beam structure of the driving beam through conservation of both orbital and spin angular momentum, producing unique harmonic beams with: (1) highly charged fractional-OAM; (2) linear polarization distribution along the light ring, where diametrically opposite points are orthogonally polarized. Our results show that OAM and SAM are not exchanged in CR-driven HHG, but conservation of both quantities separately leads to the generation of fractional-OAM harmonics. In addition, fractional-OAM beams are produced with similar divergence, thus allowing for the synthesization of structured attosecond helical beams or “structured attosecond light beams”, unique spatio-temporal field structures with promising applications in the inspection of dynamics of non-homogeneous molecular systems at the nanometric and attosecond scales.

Though the spatio-temporal properties of the fractional-OAM harmonic beams could be harnessed using macroscopic phase-matching (as it was proposed for integer-OAM harmonics^28^), their energy content could be extended up to the soft x-ray regime if CR driving beams with longer, mid-infrared^23^, or shorter, ultraviolet^53^ wavelengths were used. On the other hand, our proposal opens the route to generate EUV/soft x-ray fractional OAM beams in other scenarios, such as harmonic vortices generated in plasmas^54^,^55^,^56^, or solid targets^57^,^58^, and vortex beams generated in free electron laser facilities^59^. Finally, we note that the particular polarization and shape of the coherent EUV/x-ray beams here predicted could be applied in fields such as EUV litography^60^,^61^, surface structuring^62^, optical trapping^44^, and super-resolution imaging^63^.

## Methods

### Theory of conical refraction

The geometric optical approximation of the radius of the dark ring, *R*_0_, splitting the two characteristic bright rings of CR is the product of the crystal length, *l*, and the CR semi-angle *α*, i.e., *R*_0_ = *lα*^16^,^45^. The CR semi-angle *α* depends on the principal refractive indices of the crystal as 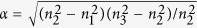, where we have assumed *n*_1_ < *n*_2_ < *n*_3_. For KGd(WO_4_)_2_ and KTP biaxial crystals typical values of *α* at *λ*^0^ = 800 nm are ≈1*6* mrad^64^. This, together with feasible crystal lengths ranging from 1–30 mm, yields CR rings in the range 16–500 μm.

The paraxial solution describing CR was derived by Belsky and Khapalyuk^41^ and later reformulated by Berry^42^. For a uniformly polarized and cylindrically symmetric input beam, the general properties of the CR beam are described by the following two components:


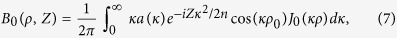



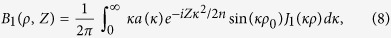


where we use cylindrical coordinates (*ρ, φ, Z*) normalized to the beam waist radius *w*_0_ and Rayleigh range *z*_*R*_ of the input beam, i.e., 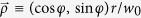, 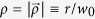 and 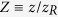, with origin at the ring center (*ρ* = 0) at the focal plane (*Z* = 0). Besides 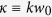, *k* being the spatial wave-vector, *n* is the average refractive index of the biaxial crystal (*n* ≈ 2 in most biaxial crystals^64^), 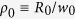, *J*_*q*_ is the *q*^*th*^-order Bessel function of the first kind and 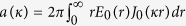 is the Hankel transform of the input beam. For a Gaussian input beam 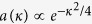 43,44. The characteristic pair of concentric bright rings of CR are found at the otherwise expected focal plane of the focused Gaussian input beam and they are preserved approximately during a distance *z*_*R*_ from the focal plane. Far from the focal plane, both light rings become wider and at 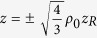 most of the light becomes concentrated on the beam axis^65^. For a circularly polarized input beam described in the basis 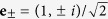 (+ for right-handed and − for left-handed circularly polarized), the electric field behind the crystal becomes:


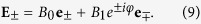


**Figure 6:**
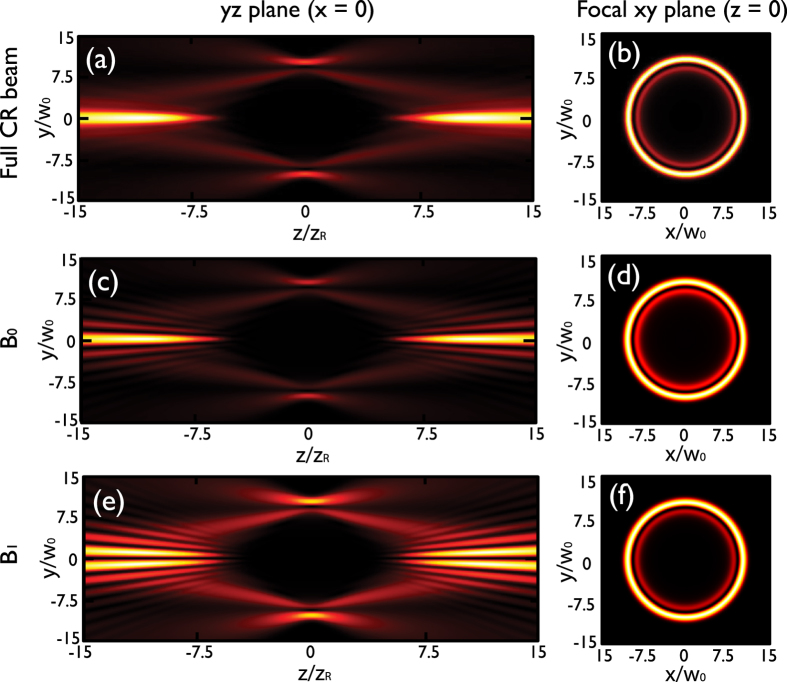
Conical refraction for a circularly polarized Gaussian input beam with *R*_0_/*w*_0_ = 10. Images (**a**,**c**,**e**) are cuts of the intensity pattern in the *yz* plane at *x* = 0 showing the space evolution of the CR beam, the *B*_0_ component, and the *B*_1_ component respectively. Images (**b**,**d**,**f**) show the transverse intensity distribution obtained at the focal plane of the CR beam, the *B*_0_ component, and the *B*_1_ component, respectively.

From Eq. (9) one can appreciate that the particular features of CR beams are a result of the interference of the two conical beams *B*_0_ and *B*_1_ described in Eqs (7) and (8). The *B*_0_ beam preserves the state of polarization of the input beam and possesses null OAM. In contrast, the *B*_1_ beam is orthogonally polarized to the input beam and carries one unit of OAM per photon, due to the spin-orbit coupling introduced by the biaxial crystal^11^,^14^. As a consequence, the global CR beam possesses 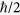 OAM per photon^11^,^12^,^13^,^14^,^15^. In fact, the *B*_0_ and *B*_1_ components can be physically filtered out from the global CR beam by means of circular polarizers, allowing for a separate observation of their free-space evolution^12^,^13^,^66^,^67^. To better visualize the shape of both components, in Fig. 6 we show density plots of the intensity distribution of the complete CR beam [panels (a) and (b)], the *B*_0_ component [panels (c) and (d)], and the *B*_1_ component [panels (e) and (f)] in the *yz* plane at *x* = 0 (first column) and in the *xy* plane at *z* = 0 (second column). As it can be appreciated, the *B*_1_ component possesses an on-axis null intensity point preserved upon propagation due to its topological charge. This null intensity point is not present in the *B*_0_ component, since its topological charge is null. In our numerical simulations we have used the top sign of Eq. (9) as the input NIR CR beam that produces the HHG.

### Theoretical method to compute high harmonic generation including propagation

We study the generation of EUV/x-ray vector beams through HHG, by performing numerical simulations that make use of an extended strong field approximation, and that include propagation through the electromagnetic field propagator^48^. We discretize the target (gas jet) into elementary radiators, and propagate the emitted field *E*_*j*_(*r, t*) to the detector,


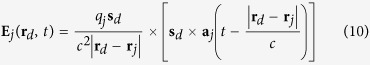


where *q*_*j*_ is the charge of the electron, **s**_*d*_ is the unitary vector pointing to the detector, and **r**_*d*_ and **r**_*j*_ are the position vectors of the detector and of the elementary radiator *j*, respectively. The dipole acceleration **a**_*j*_ of each elementary source is computed using an extension of the strong field approximation. Note that Eq. (10) assumes that the harmonic radiation propagates with the vacuum phase velocity, which is a reasonable assumption for high-order harmonics. Finally the total field at the detector is computed as the coherent addition of the elementary contributions. Propagation effects in the fundamental field such as the free charges and neutrals, group velocity matching^68^ as well as absorption in the propagation of the harmonics, are also taken into account. One of the advantages of this method is its suitability to compute high-order harmonic propagation in non-symmetric geometries. Therefore, for example, it is specially suited for computing HHG driven by beams carrying orbital angular momentum^26^,^28^,^30^, or spatially dependent polarization^33^,^36^.

In the simulations presented in this work, the laser pulse envelope *A(t*) is assumed to be sin^2^(*t*) of 2.9 cycles (7.7 fs) full-width-half-maximum (FWHM), whose amplitude (*E*_0_) is chosen to give a peak intensity at focus of 1.4 × 10^14^ W/cm^2^ at a wavelength of *λ*_0_ = 800 nm. The fundamental beam is modeled as a CR beam described by Eqs (7, 8, 9), where the beam waist radius is *w*_0_ = 15 *μ*m and *R*_0_ = 10*w*_0_. The resulting radial vector beam is focused into an argon gas jet (as depicted in Fig. 1) directed along the *x*-axis. The transverse density profile of the gas jet is modeled by a Gaussian distribution along the *y* and *z* dimensions (whose FWHM is 200 *μ*m) and possesses a constant density profile along its axial dimension, *x*, with a peak density of 10^17^ atoms/cm^3^.

## Additional Information

**How to cite this article:** Turpin, A. *et al*. Extreme Ultraviolet Fractional Orbital Angular Momentum Beams from High Harmonic Generation. *Sci. Rep.* **7**, 43888; doi: 10.1038/srep43888 (2017).

**Publisher's note:** Springer Nature remains neutral with regard to jurisdictional claims in published maps and institutional affiliations.
